# Characteristics of Academic Adaptation and Subjective Well-Being in University Students with Chronic Diseases

**DOI:** 10.3390/ejihpe10030059

**Published:** 2020-08-20

**Authors:** Rail M. Shamionov, Marina V. Grigoryeva, Elena S. Grinina, Aleksey V. Sozonnik

**Affiliations:** Faculty of Psychological, Pedagogical and Special Education, Saratov State University, Astrakhanskaya Ulitsa, 83, 410012 Saratov, Russia; grigoryevamv@mail.ru (M.V.G.); elena-grinina@yandex.ru (E.S.G.); sznnik@mail.ru (A.V.S.)

**Keywords:** academic adaptation, subjective well-being, university students, chronic diseases

## Abstract

Studying academic adaptation and subjective well-being in students with chronic diseases can help to explain psychological compensatory mechanisms and help with the development of socio-psychological support programs. It is supposed that the defining role is played by general adaptive potential, and the presence of chronic diseases results in variations in academic adaptation, which, alongside other variables, acts as a predictor of subjective well-being and satisfaction of basic needs. The sample consisted of first-year university students aged 17–26 years (mean = 19.6, SD = 2.8, 18.4% male; *n* = 419 persons, of which 34.8% with chronic diseases of various etiologies). To evaluate the components of students’ academic adaptation, we used the Academic Adaptation Scale; general adaptive potential was measured using the Multilevel Personal Adaptability Questionnaire; to evaluate subjective well-being, we used the Subjective Well-Being Scale; and satisfaction using the Life Scale. Satisfaction of basic needs was defined with the Basic Needs Satisfaction in General Scale. Students with chronic diseases demonstrated lower manifested adaptive potential, general markers of academic adaptation, subjective well-being, and satisfaction of basic psychological needs. The results showed that interrelations between various markers in students are largely mediated by academic adaptation and adaptive potential. Thus, the interconnection between adaptive potential and satisfaction of basic needs is significantly mediated by students’ academic adaptation, whereas the interconnection between chronic diseases and academic adaptation is mediated by adaptive potential. In other words, the findings support the assumption regarding the significant mediating role of these variables in subjective well-being. Cognitive, motivational, and communicative components of academic adaptation can serve as compensatory factors for experiencing subjective well-being in students with chronic diseases.

## 1. Introduction

Modern life, which is marked by deteriorations in the environment, poor nutrition quality, and chronic stress, negatively influences human health. Only 66.6% of young Russians aged 15–19 years and 68% of young Russians aged 20–24 years describe their health as “good”; 15.8% aged 15–19 years and 16% aged 20–24 years describe their state of health as “satisfactory”; and 1.1% and 1.7% of the respondents evaluate their health as “poor” or “very poor,” respectively [1]. According to data from the Ministry of Health of the Russian Federation, which were presented at the All-Russian Forum on Public Health, about 50% of the Russian population has chronic disease [2]. Currently, there are different approaches to understanding the phenomenon of chronic diseases. Thus, Hale wrote, “...the state of chronic illness involves impairment of physiological processes that restricts activity and function, even if the underlying impairment is poorly understood and intangible” [3] (p. 8). All chronic diseases are characterized by a significant decrease in the body’s endurance. Chronic disease differs from other disorders and diseases in that it affects global functions, both physical and cognitive, and is not localized to a specific organ, being unstable and fluctuating. Health disorders can influence human psychology and negatively impact the human ability to adapt. 

Significant changes are occurring in both the lifestyle and activities in the course of study at higher educational institutions. When a person enters university, they undergo changes in their life situation, such as moving to a new place or changes in routine commute, inclusion in a new social environment, changing forms of education, and mental stress. The necessity to adapt to educational conditions, the new collective, and professional activity requires mobilization of all resources of the human system. It is also important to create conditions for equal opportunities for education regardless of the presence or absence of diseases within educational environment. 

However, research on the academic adaptation of students with chronic diseases clarifying the psychological compensatory mechanisms of their adaptation is still insufficient. This is why versatile analysis is needed on adaptation of students with chronic diseases compared to students without chronic diseases, as well as comparative analyses of factors that characterize attitude to life in general, which is expressed through their subjective well-being. This knowledge will assist with the development of programs for adaptation and the provision of socio-psychological support for students with chronic diseases.

### 1.1. Academic Adaptation as a Health Factor

Academic adaptation is the process and result of student adjustment to the educational environment, including the system of interpersonal relations in education, educational activities, and educational space, which characterizes the experience of a dynamic balance between the individual and the educational environment. Academic adaptation of university students has been studied in its interconnection with academic performance and personal-emotional adaptation [4], academic self-control [5], nature of relationships within the student environment [6], characteristics of the family situation [7], and ethical convictions [8], which are important components of the academic environment and, particularly, the accepted norm of academic honesty [9], as well as many other variables. Many studies focused on the academic adaptation of international students in host universities all around the world [6,10,11,12]. This is a relevant problem due to increasing academic mobility. These investigations allowed for tracing students’ characteristics, which are important for their academic adaptation, and for defining the connection between adaptation and internal personal resources, as well as the connection between adaptation and general psychological and physical health. Thus, recent studies found that academic self-efficacy, social support, and low levels of perceived discrimination predict both psychological and academic adaptation of Chinese students in English-speaking countries [12]. These variables are relevant for students that attend university in their home country. Despite prejudices in the student environment (based on the signs of otherness, i.e., physical or social) being manifested to a lesser degree than in other environments, the projected discrimination lowers young people’s adaptive potential [13]. 

An important condition for students’ academic adaptation at the beginning of their academic career at university is an orientation that defines the purpose of their study using a wide range of learning strategies and level of academic involvement [14]. Transition from a school educational environment to university presupposes adaptation to a new social and spatial environment and to a new academic environment, which does not have such strict control but simultaneously requires academic skills, which have not yet been fully developed in former schoolchildren. A change in learning strategies is required, which is often a dramatic process and affects not only academic performance but also university students’ psychological state [15]. However, academic adaptation of university students is important for productive learning. Thus, a number of studies showed that academic performance is predicted by successful adaptation to university (emotional and personal adaptation) and pre-university performance [4].

Students that do not succeed can demonstrate perseverance driven by desire to complete their course of studies and to live up to expectations and thereby pursue academic adaptation [16]. Researchers reported that adaptation of such students is mostly associated with changes in their own learning habits and prioritization, as well as support from family and friends [16] (p. 90). A study regarding effects of academic stressors on mental health [17] established that evaluative stress is the strongest type of stress, and strong social connections at university are the health factor. In other words, inclusion in a student group, as one of the indicators of academic adaptation, acts as an important factor of students’ mental health. Zimina et al. [18] emphasized the significant influence of the state of both physical and psychological health on adaptive resources of the human system. Thus, the authors found that adaptation mechanisms in students are reduced, as indicated by the low level of psychological well-being, low level of stress resistance, reduced activity, negative attitude toward themselves, and dissatisfaction with the circumstances of their lives. Despite available data on a possible interconnection between academic adaptation and subjective well-being, there has been no specific study of this issue. 

### 1.2. Academic Adaptation of Students with Chronic Diseases

The academic adaptation of students with chronic diseases is burdened by a number of problems that are not only related to their state of health, but also to their attitude to their physical condition, state of health, the need to constantly monitor a number of its parameters, as well as identity with good health or ill health.

A special group, which has so far not attracted any close attention from researchers and practitioners, is represented by students with chronic diseases, despite their impressive number [19,20,21]. Students with chronic diseases are often not viewed as subjects in need of directed psychological support. Researchers [22] noted that chronically ill people often experience a dissonance between “healthy” identification and the need to prove the status of their ill health at university to receive academic support, which causes contradictions in their adaptation to the educational environment. The peculiarities of one’s state of health are interconnected with the mental and psychological states of an individual [23,24], which may affect the success of their academic adaptation and experience of subjective well-being. Recognizing individual characteristics, like health status and psychological state of each person with a chronic disease, is necessary to acknowledge the possible impact of the disease on their psychology and the formation of some aspects of activity that can occur in people with various diseases, including restrictions in the exercise of their requirements. Thus, according to data provided by Sidorov et al. [25], when examining students with chronic diseases, manifested signs of psychosocial maladaptation are observed in 77% of cases. Such students are characterized by a tendency to depression and anxiety, timidity, restraint, low activity, low self-esteem, and a noticeable dissonance in personal relationships. In addition, male students are more at risk of academic maladaptation [26]. Gavrilova [27] revealed that motivation for success in female students with chronic diseases is slightly lower than in healthy students, whereas it is better expressed in young males with chronic disorders. Having analyzed the psychological characteristics of students with various chronic diseases, Gazova and Khushtova [28] reported that students with chronic diseases are worse at coping with problem situations; they choose non-productive coping strategies (humility, confusion, dissimulation, ignoring), and do not use cognitive coping strategies. These features, in our opinion, do not provide grounds for the formation of a negative stereotype of students with chronic diseases, but allow us to recognize the difficulties in their academic adaptation and the need to implement support measures in the process of obtaining education. Notably, admission to university requires significant efforts from a person with a chronic disease, including updating their existing abilities, and overcoming many barriers, which can be considered as a significant personal achievement and a step toward social integration. This position is consistent with the positive model of disability [29], which is associated with the refusal to understand the phenomenon of health disorders as a personal tragedy, and the emphasis on positive aspects of social identity. However, in the future, in the process of obtaining education, students with chronic diseases may face various difficulties, such as the inaccessibility to various aspects of the educational environment, which may negatively affect their ability to adapt and their psychological well-being. Hughes et al. [30] noted the presence of special needs in students with chronic diseases, for which many of them seek help from disability support services. As possible prerequisites for difficulties in adapting to university, most of them note less violations of physical, intellectual, or sensory health, but more report emotional and psychological problems. Njoku [21] stated that such students need educational support; however, it is not provided in the traditional educational model. Their unfavorable position is associated with reasons such as the negative attitude of teachers, breaks in study, as well as insufficiency of their own resources. Hutcheon and Wolbring [31] considered it necessary to thoroughly study the experience of students with different abilities. The existing abilities and their development, not the possible shortcomings of students, should be the basis for analyzing the policies in the field of higher education, ensuring access to adaptive technologies for students with diverse needs. 

According to Shiu [32], understanding educational needs of students with chronic diseases is required to ensure that they have equal educational opportunities. However, as noted by L. Royster and O. Marshall, given the specificity of such students, in most cases, they do not identify themselves with disabled people, but they may also experience difficulties with learning and academic adaptation. To minimize this problem, the Chronic Illness Initiative (CII) program, implemented at the DePaul University (USA), was proposed. It includes various aspects for increasing student self-efficacy, social support, academic support, and teacher training [33]. The implementation of the program includes such aspects as the organization of distance education for students with chronic diseases who may experience difficulties in visiting an educational institution. According to the authors, 80% of students with chronic diseases used the online option when completing the educational program. A significant aspect involves working with teachers and staff on issues related to chronic diseases to form an adequate attitude to students. There is also support for students, including issues related to health, living conditions, administrative issues, financial support, employment, etc. Much attention is paid to the social integration of students with chronic diseases, regardless of whether they attend classes or study online. As a result of the implementation of this initiative, positive trends have been observed in the education of students with chronic diseases. This is manifested by a decrease in the number of students who are expelled, their higher academic success, and their increased educational activity. These circumstances allowed the evaluation of the capabilities of the Chronic Illness Initiative in optimizing the academic adaptation of students with chronic diseases. Thus, the researchers noted the possible presence of psychological disorders in people with chronic diseases, the need and ability to overcome difficulties in the process of self-realization in various fields, and the special educational needs of students with chronic diseases. Investigations in this problem field have not been complete, but rather fragmentary. 

### 1.3. Subjective Well-Being and Health 

The problem of subjective well-being in connection with health was posed by psychologists when the first empirical studies of this phenomenon were conducted. It is primarily related to the subjective well-being being defined as a lack of ill-health (disease) [34], as well as to subjective well-being being perceived as psychological health in humanistic concepts [35]. The notions of a healthy person’s chances of satisfying needs being high and of a person’s activities contributing to achievement of goals, which collectively create conditions for experiencing satisfaction and happiness, can be traced in many studies. Attempts have been made to correlate eudemonistic and hedonistic well-being with biological body parameters [36], which proved to be successful. Scientists revealed the close interconnections between biomarkers (neuroendocrine, immune, and cardiovascular) and markers of eudemonistic well-being; moderate interconnections were revealed between biomarkers and hedonistic well-being [36]. Argyle generalized that there is a two-directional relationship between health and well-being: Health is the reason for happiness (sometimes subjective health is more closely interconnected with well-being), and well-being influences health through activation of the immune system, which is caused by good mood [37]. The recent studies of L.I. Wasserman et al. support this statement. In the case of diseases that obviously threaten a person’s life, personal psychological maladaptation occurs; withdrawal into illness and withdrawal from fight are observed; all of the above reflect the negative tendencies in subjective well-being. Next, prevalence of negative emotions together with the specific type of cognitive-affective organization condition’s actualization of somatization processes in psychosomatic and somatopsychic correlations [38]. Thus, the psychosomatic circle appears; one of its psychological features is negative perception and low assessment of quality of life and one’s own well-being. A number of recent studies proved this to be right. Therefore, analyzing the relationship between internal positive personal resources and indicators of happiness in endocrinology patients, A.N. Samsonova, O.Yu. Khabarova, T.V. Yakimova [39] found that average (40.6%) and low (37.5%) indicators of happiness prevail. T.V. Kaurova and G.L. Mikirtichan [40] showed that all components of the quality of life, which is closely associated with the concept of subjective well-being, are significantly lower in adolescents and young people with chronic dermatoses than in their healthy peers. Among adolescents with impaired renal function, higher rates of egocentricity were observed, which were less critical; their self-image was quite superficial and poorly differentiated, and low self-esteem was combined with high level of aspirations. Psychological ill-being of such children is evidenced by significant gaps between assessing their current state, particularly state of health, and their desired state [41]. Uncertainty, which is characteristic of people with chronic diseases, determines actualization of stress, emotional maladaptation, and activation of psychological protection mechanisms [42]. Finally, satisfaction with quality of life is lower in patients with cicatricial deformities of the face and neck [43]; a decrease in the quality of life in patients with motor disorders of various etiologies was also reported [44].

Additionally, personal characteristics are important as they form attitude toward health and disease, as well as mood; all of these predict variations of subjective well-being. Later, Diener pointed out that adaptation to conditions is not always full and that sometimes circumstances have a huge impact on subjective well-being, but high levels of well-being positively influence human health [45]. In other words, problems related to adaptation to a change in situation can significantly influence achievement of subjective well-being. However, adaptation is never complete because a situation change constantly creates certain stress related to the necessity to adapt.

Recent studies [46] showed that presence of a chronic disease not only negatively influences self-esteem with one’s physical and psychological state, but also negatively influences subjective well-being as a whole. Researchers identified the relationship between subjectively perceived health and subjective well-being. Notably, subjective well-being of chronically ill students depends on social support regardless of the stress they experience [47]. Social support is a factor that influences evaluation of personal resources as sufficient for adaptation. An important aspect of the interconnection between health and subjective well-being is the socio-ecological environment, which can explain where the differences lay, e.g., in college students [48]. Environmental factors play an important role in reducing anxiety and increasing trust. This role could be partly played by the university educational environment, which could create an atmosphere of trust and stability by implementing a strategy of equal opportunities for students with chronic diseases. This would help facilitate their adaptation to university and, therefore, contribute to their subjective well-being.

The purpose of the study was to investigate characteristics of academic adaptation and subjective well-being of students with chronic diseases, including (1) a comparative analysis of the components and general indicators of academic adaptation, as well as adaptive potential of students with chronic diseases and healthy students; (2) a comparative analysis of indicators of subjective well-being (happiness and life satisfaction) and satisfaction of basic needs (in autonomy, competence, and relatedness with other people) in students with chronic diseases and healthy students; and (3) based on structural modeling, testing the hypothesis regarding the role of academic adaptation and adaptive potential in subjective well-being and basic needs satisfaction in students, considering the presence/absence of chronic diseases.

We assumed that the academic adaptation and subjective well-being of students with chronic diseases have certain features in comparison with that of healthy students. The academic adaptation and adaptive potential of students play a mitigating role in the subjective well-being and satisfaction of basic needs of students due to the presence/absence of chronic diseases.

## 2. Materials and Methods 

### 2.1. Sample

First-year university students aged 17–26 years (mean (M) = 19.6, SD = 2.8 years) participated in this study (sex: 18.4% men and 81.6% women). Of the *n* = 419 participants, 34.8% had chronic diseases of various etiologies (21.2% vision disorders, 8.2% musculoskeletal disorders, 2.1% emotional-volitional disorders, 8.4% combined disorders, 9.8% other, which correlates with the “norm” of today and the results of other studies [18,19]); 373 were single (89.4%), nine married (2.2%), and 27 other (6.4%). Before entering university 5.7% of students lived in a metropolis, 42.3% in a city, 38.8% in a town, and 13.6% in a village. All subjects provided their informed consent for inclusion before they participated in the study. The experimental studies were performed in accordance with the Ethical Standards (2000) and were approved by the local research Ethics Committee of the Saratov State University (faculty of psychological, pedagogical, and special education).

### 2.2. Design of the Study

The study was designed as follows: First, the socio-demographic parameters of students with chronic diseases and students that had not been diagnosed with any diseases were analyzed. A comparative analysis was conducted of the components of academic adaptation and its integral indicator, the overall adaptive potential, characteristics of subjective well-being (life satisfaction and happiness experience), and satisfaction of the basic needs of students (for students with and without chronic diseases). Finally, structural equation modeling (SEM) was used to test the hypothesis about the role of academic adaptation and adaptive potential in subjective well-being of students.

### 2.3. Measurements

To identify socio-demographic markers, we developed a questionnaire to capture data regarding age, sex, diseases, place of residence before entering university, and level of income in the family. 

To assess the components of students’ academic adaptation, we used the Scale of Students’ Academic Adaptation (Shamionov, Grigoryeva, Grinina, Sozonnik). The scale contains 44 points, each of which is evaluated by the respondent according to a Likert scale (from 1 to 5 points). Seven scales are obtained as a result of filling out the questionnaire: Personal, emotional-evaluative, cognitive, motivational, psycho-physiological, communicative, and integral assessment of academic adaptation. The scale demonstrated good psychometric indicators: Cronbach’s α = 0.93 when the item was removed; the normality of distribution of the integral assessment distribution check produced an acceptable result (*Z* = 0.701; *p* = 0.71). 

To study the adaptive capabilities of an individual based on the assessment of certain psycho-physiological and socio-psychological characteristics, we used the multilevel personal questionnaire called Adaptability (Maklakov, Chermyanin, 2006). The technique includes 165 points with which respondents agree or disagree. Four major scales are obtained based on the key: Behavioral regulation, communicative potential, moral normativeness, and personal adaptive potential (all scales are regressive), as well as the reliability scale. The scales had sufficient reliability, Cronbach’s α = 0.81–0.88. 

To assess the degree of needs satisfaction in autonomy, competence, and relatedness, we used the Basic Needs Satisfaction in General Scale [49] adapted for the young Russian population by R.M. Shamionov. The scale contains 21 points and three subscales (autonomy, competence, and relatedness). The scales had sufficient reliability, Cronbach’s α = 0.77–0.82. 

The assessment of subjective well-being included two major parameters: Satisfaction with life and experiencing happiness. The subjective happiness scale was constructed by S. Lyubomirsky and H. Lepper (1999) and adapted by D.A. Leontiev and E.N. Osin (four-item scale). The scale has acceptable reliability level happiness (H) with Cronbach’s α = 0.78. The Satisfaction with Life Scale by E. Diener, R.A. Emmons, R.J. Larsen, S. Griffin (1985) and adapted by D.A. Leontiev and E.N. Osin (five-item scale) had a good reliability level satisfaction with life (LS) with Cronbach’s α = 0.88. Subjective well-being scales are assessed according to a seven-point scale depending on the agreement/disagreement with the statement, or manifestation/lack of manifestation of the trait. 

### 2.4. Statistical Analysis

To process primary data, we used the statistical software package IBM SPSS Statistics + PS IMAGO PRO, which includes AMOS software, which can be used for modeling with structural equations. 

First, the scales were checked for internal consistency by using the Cronbach’s alpha coefficient and the data were checked for normality of distribution. Then, the socio-demographic data were studied with the help of descriptive statistics (depicting in the averages, standard deviations and percentages). After that, the average values in two groups (with and without chronic diseases) were compared according to the students’ criterion. All the previous indicators meet the requirements for the usage of this criterion.

In the next stage, we conducted a simulation procedure using AMOS for structural equation modeling. This program helped us to confirm the preliminary hypotheses, to establish the directions of relationship and the criteria for model acceptance were set (chi-square (CMIN), degrees of freedom (df), comparative fit index (CFI), adjusted goodness-of-fit index (AGFI), goodness-of-fit index (GFI), root mean square error of approximation (RMSEA)). In accordance with the model requirements [50], the statistical significance of all regression coefficients, covariance between variables and variances were verified. Next, we analyzed the calculation results, detected the direct and indirect effects, coefficient of determination (R^2^) as a measure of the proportion of the variance of the dependent variable about its mean which is explained by the independent variables (it is indicated in the image top right number on each dependent variable).

## 3. Results

Table 1 presents the socio-demographic and academic performance parameters at the university for individuals manifesting chronic diseases without any serious health disorders and the general sample.

Table 1 shows that parameters of distribution of students according to age and sex were approximately the same. We found significant discrepancies in the distribution of parameters of residence before starting university and income. Individuals with chronic diseases have slightly more excellent marks (53.4% vs. 42.5%), and, correspondingly, fewer good marks (33.6% vs. 42.9%). Notably, the average academic success of students, which is assessed based on the results of the examination period, had practically no discrepancies (*t* = 1.73, *p* < 0.08). 

Table 2 shows that the integral assessment of academic adaptation in students with chronic diseases was significantly lower than in students without chronic diseases. Psycho-physiological, emotional-evaluative, and personal components of academic adaptation contributed to these differences. For all these components, students with chronic diseases demonstrated lower figures than their healthy peers. Assessments of psycho-physiological component varied the most. 

The personal component of academic adaptation was also lower in students with chronic diseases (Table 2). Students with chronic diseases were less able to organize space around themselves in the learning process, set fewer educational goals, found it difficult to fix material in lectures, planned their educational activities less often, and so on. The disease and physical difficulties in the learning process do not provide a full opportunity to focus on academic achievements and the desire for better organization of their educational activities. Accordingly, the emotional and evaluative component of academic adaptation was also lower (Table 2): Students with chronic diseases were less satisfied with the process and results of training at the university, relationships with teachers, the convenience of academic facilities, the information environment of the university, etc. To a lesser extent, students with chronic diseases expressed positive emotions in the educational process compared to others. 

Overall assessment of adaptive potential in the group of healthy students was significantly higher than in the group of students with chronic diseases (Table 3). Behavioral self-regulation was higher due to higher self-esteem, adequate assessment of the surrounding reality, and a good level of neuro-psychic resistance [51]. 

The results obtained using two methods (Scale of Academic Adaptation of Students and the Multi-Level Personal Questionnaire (MLO) “Adaptability”) were consistent. Students with chronic diseases were found to be more sensitive to the difficulties of the educational process. Due to the disease and the load on the physical and neuropsychic structures of the body, they have limited opportunities for self-organization and overcoming adaptive difficulties, which negatively affect the adaptation to the conditions of education.

Table 4 shows that students with chronic diseases had a lower level of satisfaction with almost all basic needs, except for the need for relatedness (ability to connect with other people). Their satisfaction with the need for autonomy was significantly lower than in other students, which is conditioned by their certain dependence on the environment and lack of confidence in their independent actions. This, in turn, can be the consequence of negative experiences with independent actions, as well as learned helplessness. In this regard, their satisfaction with competence was satisfied to a lesser degree. 

Experiencing happiness and general satisfaction with life were less manifested in students with chronic diseases compared to other students (Table 4). This may be due to recognition of their differences from healthier people, experiencing physical distress, self-regulation difficulties due to health limitations, and other factors mentioned above. 

**Figure 1:**
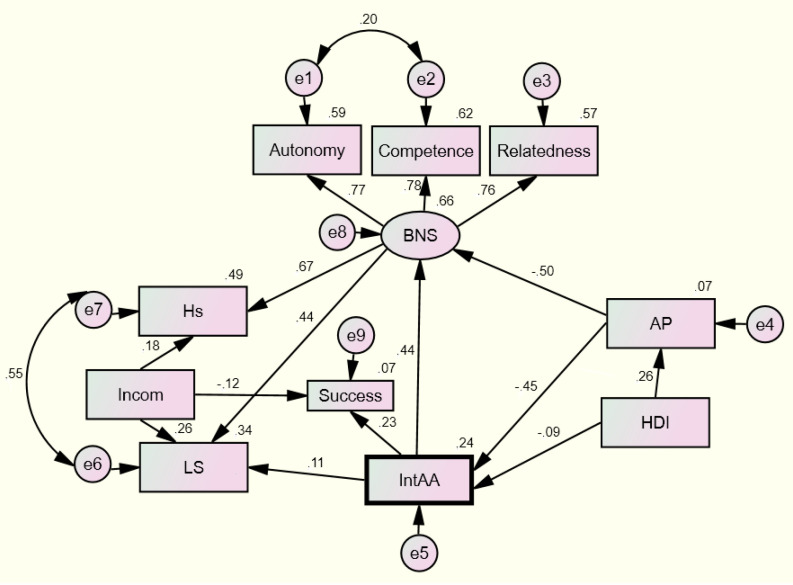
Roadmap for parameters of academic mobility and subjective well-being. Hs, happiness; LS, life satisfaction; BNS, satisfaction of basic needs; Income, level of income in the family; Success, Educational success; IntAA, Academic adaptation; AP, Adaptive potential; HDI, Presence/absence of chronic diseases.

Next, we tested the hypothesis about the direction of connections from students’ academic adaptation to satisfaction of basic needs and satisfaction with life from adaptive potential to satisfaction of basic needs and academic adaptation (Figure 1, Table 5). The model complied with the initial data. All evaluated parameters were statistically valid at the *p* < 0.05 level. This model explained up to 24% in the variation in experiencing happiness and 34% of the variation in experiencing satisfaction with life. The model showed that the major contribution to academic adaptation was by adaptive potential (regressive scale) and the presence/absence of chronic diseases. In both cases, presence of chronic disorder was a factor affecting the decrease in numbers.

## 4. Discussion

From the obtained results, we found that the number of male and female students was distributed approximately equally in both groups, which corresponded to general sample indicators. Distributions by place of residence before entering a university were notable. Among healthy students, more than twice as many healthy students were from the countryside and half as many from metropolises than chronically ill students. These data may indicate that chronic diseases develop less intensively under rural conditions, which are more environmentally friendly. However, this finding may be the result of better medical care and services in cities, due to which chronic diseases are detected at an earlier stage. In other words, better medical diagnostics in a large city or metropolis contribute to a greater number of detected diseases in cities compared to rural settlements. The increase in the number of chronic diseases in cities compared to rural areas is also influenced by factors such as the higher risk of spreading viral diseases in the city, a higher level of competition that increases psychological stress, amongst others.

Comparative analysis of academic adaptation of students with chronic diseases and healthy students revealed a number of findings. The difference in markers in terms of the emotional-evaluative component manifested in lower satisfaction of students with chronic diseases with the spatial-subject and social components of university educational environment, which may be due to increased educational environment requirements and sensitivity of the body and psyche to external influences in chronic diseases [30]. 

Markers of the personal component of academic adaptation in students with chronic diseases were significantly lower than in students without chronic diseases. In the former, students coped worse with self-organization in the academic process worse and were less focused on self-changes and had less desire to plan and achieve academic goals. They had a less manifested ability to organize their living space in the course of the academic process. Restrictions on the ability of students with chronic diseases to self-organize and organize the living space around them, which affect the redistribution of their internal reserves from academic achievements to combat ill health, create problems with having a more focused organization of the educational space and with identifying physical and subject barriers that cause difficulties in interacting with students with chronic diseases within the educational environment.

The internal reserves of the system and psychology of students with chronic diseases are limited due to re-distribution of energy aimed at struggling with the physical problem; they require compensatory and primarily external factors and reserves to increase academic adaptation [52,53].

The approximately equal cognitive, motivational, and communicative components of academic adaptation in groups of students with and without chronic disorders (Table 2) indicated the possibility of achieving a good level of academic adaptation due to their desire to acquire knowledge and well-developed educational competencies, which are primarily associated with processing and storing large volumes of information, correlating it with existing knowledge, and the ability to design difficult learning situations and solutions. Absence of differences in the level of development of the communicative component of academic adaptation in the two groups also testified to the possibility of students with chronic diseases equally interacting with the social environment and other students, openly expressing and proving their point of view, cooperating with others, fulfilling tasks, and presenting themselves to others. Perhaps, cognitive, motivational, and communicative components, in the course of their further development, act as compensation for the less-manifested psycho-physiological, emotional-evaluative, and personal components of academic adaptation.

Comparative analysis of adaptive capabilities markers showed the absence of differences in the level of manifestation of communicative potential, which, once again, confirmed the inclusion of students with chronic diseases in social relationships as being a par with other students.

Moral normativity and perception of moral standards of behavior accepted in the society, as well as understanding of the requirements of the immediate social environment (Table 3), were expressed at approximately the same level. This indicates that chronic disease presence does not have any impact on their deep personal structures. These data are consistent with a number of studies that reported that students with chronic diseases have the required personal resources to establish relationships with others and to communicate but are less able to cope with adaptation difficulties [21].

Finally, comparison of the mean indicators of subjective well-being (happiness and satisfaction with life) and satisfaction of the basic needs of students with chronic diseases and healthy students allowed us to establish the presence of significant differences on all scales, except satisfaction of the need for relatedness with other people. The absence of differences in personal characteristics determined by social relations was a theme throughout the entire study [29]. This means that students with chronic diseases, alongside healthy students, fulfill their relationships in society and, due to this, they can compensate for a number of objective difficulties associated with learning and fulfilling other needs that they have. Despite, for some researchers, subjective health being a more important indicator of well-being [46], we state that students with chronic diseases are characterized by less-manifested subjective well-being. Overall satisfaction with life and its various aspects is an important indicator of adaptation, including academic adaptation. The low indicators of satisfaction of basic needs indicated a problem with the lack of equal opportunities for students with chronic diseases and other students in the educational process. Compensation for these different opportunities can be partially realized by reorienting students with chronic diseases to other needs, such as fulfilling the need for communication, creativity, etc. The university also needs to consider the special needs of students with diseases, monitor these needs, and organize the educational environment with these needs in mind. Chronic diseases are more long-term predictors of less-manifested subjective well-being, which means that we need measures of socio-psychological support for students that would contribute to formation of an attitude toward one’s life as prosperous according to the criteria of an actual life situation. In addition, the remaining prejudices against people with disabilities [13] create subjective barriers regarding equal opportunities for students with chronic diseases. The university educational environment should be organized considering elimination of physical as well as socio-psychological barriers.

Based on the SEM, we designed a model explaining about one-quarter of the variation in the academic adaptation of students. This model indicated the significant role of adaptive potential and chronic diseases in the determination of academic adaptation. In terms of indicators of subjective well-being, academic adaptation also acts as a determinant, as does satisfaction of basic needs. This result is consistent with data previously reported by us [54] and other researchers regarding the influence of the process of partial (local) adaptation on life satisfaction under different conditions and prediction of well-being through basic needs [45]. The direct causal relationship between academic adaptation and basic needs’ satisfaction can be seen from this model. This prediction does not seem accidental, since academic adaptability means a comfortable relationship with others, acquisition of learning methods, and self-consistency, which determine satisfaction of basic needs for relatedness, competence, and autonomy. These connections were confirmed by other studies that established the importance of relatedness [17], acquiring one’s own teaching methods [14], and autonomy [15] for various adaptation characteristics.

An important aspect of this model is that chronic diseases are an influential reducing factor in both academic adaptation and adaptive potential. This finding is fairly well represented in clinical psychology studies [28,46]. Finally, the model confirmed the orientation of the relationship from family income to life satisfaction and experiencing happiness, which is consistent with studies reporting that income is a subjective well-being factor in poor countries [55,56]. The direct interconnection between academic adaptation and academic performance of university students was also visible from the model, which is consistent with the previously mentioned studies by Spanish psychologists who found that academic and personal-emotional adaptation are direct predictors of academic performance [4]. The designed model also allowed the definition of the special mediating role of academic adaptation and adaptive potential. Thus, academic adaptation is a mediator of the connection between adaptation potential and satisfaction of students’ basic needs as it reduces its causal connection, and adaptation potential acts as a mediator of the connection between chronic diseases and academic adaptation. In other words, the assumption of a significant mediating role of these variables in subjective well-being is confirmed. Finally, the model confirms the causality of adaptation for satisfying basic needs and satisfaction with life, which was noted by Diener [45].

The research results raise the question of finding mechanisms for academic adaptation of students with chronic diseases that would allow them to use the potential of the educational environment to improve their self-organization and to find opportunities for their emotional support. The search for these mechanisms involves three directions: Teaching students the skills of self-regulation and self-organization in the process of academic adaptation (for example, during special training courses, socio-psychological trainings, tutor support, etc.); medical rehabilitation and maintenance of somatic health; and administrative measures to organize the educational space and facilitate the activity of students with chronic diseases (for example, an individual training plan, rest rooms, differentiated requirements from teachers, etc.)

## 5. Conclusions

Academic adaptation of students with chronic diseases has its own specifics compared with the academic adaptation of healthy students. This can be observed through low indicators of their psycho-physiological, emotional-volatile, and personal (regulatory) components, as well as preservation of cognitive, motivational, and communicative components. This specificity leads to an overall lower indicator of academic adaptation in students with chronic diseases. 

Students with chronic diseases have less of an ability to demonstrate behavioral self-regulation and general adaptive potential than other students. Nevertheless, students with chronic disorders, similar to their healthier peers, are included in social interactions in the educational process due to sufficient academic motivation, desire to learn, and the ability to design a complicated academic situation and to find the solution. 

Experiencing happiness and subjective well-being in students with chronic diseases are lower than in other students due to lower level of satisfaction of the need for autonomy and competence. 

As a result of structural modeling, we tested the hypothesis about the mediating role of academic adaptation and adaptive potential in determination of students’ subjective well-being. Presence of chronic diseases is a factor influencing adaptation. We found the cognitive, motivational, and communicative components of academic adaptation of students with chronic diseases remain well developed. 

## 6. Limitations

The limitations of this study are related to a number of circumstances. First, this research was comparative and descriptive. We did not distinguish between students with specific disease diagnoses. There may be significant differences between students with different diagnoses, making it difficult to adapt academically. In future studies, this issue should be studied. Another aspect is the subjective (declarative) nature of many issues, due to the study raising questions about the subjective attitudes of students to their psychological state and adaptation at the university. The questions of including whether the diseases are congenital or acquired and the reason for the limitations faced or discovered were also not considered. For further research, it would be appropriate to ask questions about self-assessment of the current state of health, fatigue, sleep quality, bad habits, physical activity, and specific difficulties experienced in the process of adaptation at the university. 

## Figures and Tables

**Table 1:** Socio-demographic information and parameters of academic performance and health disorders.

	With Chronic Disease (*n* = 146)		Without Chronic Disease (*n* = 273)		Total (*n* = 419)	
	%/Mean	SD	%/Mean	SD	%/Mean	SD
**Age**	**19.94**	**(3.83)**	**19.41**	**(2.04)**	**19.60**	**(2.80)**
**Sex**						
Male	17.1%		19%		18.4%	
Female	82.9%		81%		81.6%	
**Residence**						
Village	7.5%		16.90%		13.60%	
Town	39.7%		37.50%		38.30%	
City	44.5%		41.20%		42.30%	
Metropolis	8.2%		4.40%		5.70%	
**Income**	**1.86**	**(0.75)**	**2.14**	**(0.42)**	**2.05**	**(0.04)**
Significantly below average	4.8%		1.50%		2.60%	
Below average	19.2%		10.00%		13.20%	
Average	63%		64.60%		64.00%	
Above average	11%		20.70%		17.30%	
Significantly above average	2.1%		3.30%		2.90%	
**Examination period (in student’s perception)**	**4.4**	**(0.71)**	**4.28**	**(0.43)**	**4.32**	**(0.04)**
Mostly 3 (satisfactory marks)	13%		14.70%		14.10%	
Mostly 4 (good marks)	33.6%		42.90%		39.60%	
Mostly 5 (excellent marks)	53.4%		42.50%		46.30%	
**Total**	34.8%		65.2%		100.0%	

**Table 2:** Components and integral assessment of academic adaptation in students with/without chronic diseases.

Components of Academic Adaptation	Without Chronic Disease (*n* = 273)		With Chronic Diseases (*n* = 146)		Student’s *t*-Test	
	Mean	SD	Mean	SD	*t*	*p*
Personal (self-organization)	5.43	0.94	5.23	1.02	–2.01	0.05
Emotional-evaluative	5.36	1.02	5.07	1.11	–2.59	0.01
Cognitive	5.40	0.88	5.38	0.97	–0.12	0.90
Motivational	5.76	1.19	5.55	1.28	–1.65	0.10
Psycho-physiological	4.48	1.15	3.95	1.06	–4.71	0.00
Communicative	5.45	0.88	5.40	0.96	–0.53	0.60
Integral assessment of academic adaptation	31.91	4.13	30.54	4.37	–3.11	0.00

**Table 3:** Components and general assessment of adaptive potential in students with/without chronic diseases (based on A.G. Maklakov and S.V. Chermyanin’s technique [50]).

Parameters of General Adaptation	Without Chronic Disease (*n* = 273)		With Chronic Diseases (*n* = 146)		Student’s *t*-Test	
	Mean	SD	Mean	SD	*t*	*p*
Behavioral regulation	36.24	16.43	30.25	14.45	3.55	0.00
Communicative potential	14.23	5.38	13.55	4.83	1.23	0.22
Moral normativeness	7.78	3.27	8.03	3.02	–0.75	0.45
Personal adaptive potential	57.64	22.06	51.14	19.38	2.80	0.01

**Table 4:** Basic needs satisfaction, happiness, and satisfaction with life in students with/without chronic diseases.

Markers of Needs’ Satisfaction and Subjective Well-Being	Without Chronic Diseases (*n* = 273)		With Chronic Diseases (*n* = 146)		Student’s *t*-Test	
	Mean	SD	Mean	SD	*t*	*p*
Autonomy	5.19	0. 86	4.96	0.93	–2.49	0.01
Competence	4.78	0. 83	4.57	0.88	–2.34	0.02
Relatedness	5.20	0.85	5.05	0.82	–1.69	0.09
Happiness	5.17	1.09	4.58	1.22	–4.87	0.00
Satisfaction with life	4.94	1.19	4.29	1.22	–5.29	0.00

**Table 5:** Fit indices for the model.

Model	χ2	df	χ2/df	*p*	CFI	AGFI	GFI	RMSEA	PCLOSE
Indices	30.198	28	1.08	0.354	0.999	0.972	0.986	0.014	0.991
